# Supplementary material for: Development and organization of the retinal orientation selectivity map
Source: Nat Commun. 2024 Jun 6;15:4829. doi: 10.1038/s41467-024-49206-z (PMC11156980; doi:10.1038/s41467-024-49206-z)
Supplement: Supplementary file 1 — Supplementary Information [file 41467_2024_49206_MOESM1_ESM.pdf]

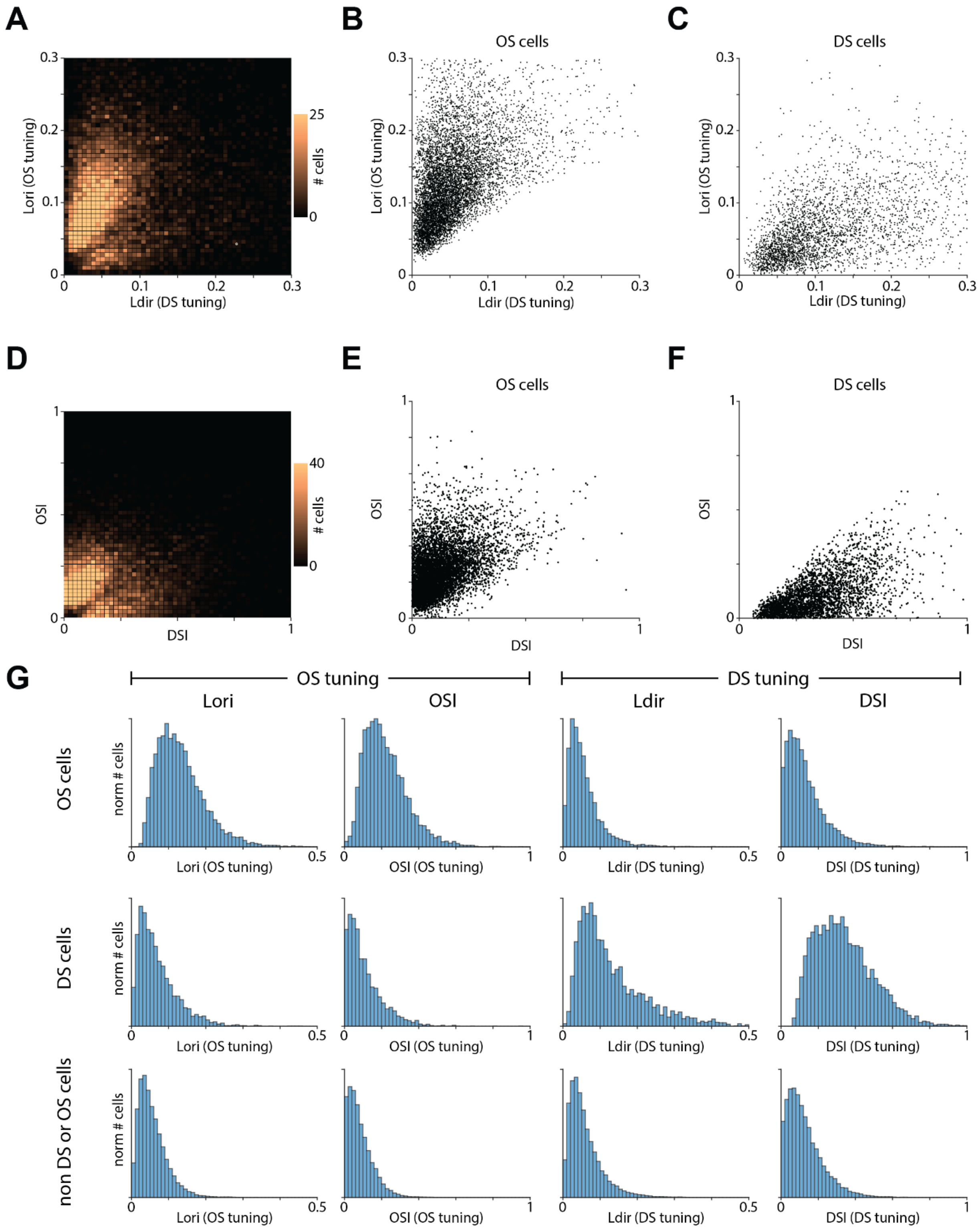
A
B
C
D
E
F
G
OS cells
DS cells
Lori (OS tuning)
Ldir (DS tuning)
cells
25
40
OSI
DSI
OS tuning
DS tuning
Lori
OSI
Ldir
DSI
non DS or OS cells
norm # cells
OSI (OS tuning)
DSI (DS tuning)

1

Supplemental Figure 1 – Vector space and index calculations lead to similar identification of orientation and direction selective retinal cells.

A. Heatmap depicting orientation tuning (L_{ori}) vs direction tuning (L_{dir}). This is the same heatmap depicted in figure 1C. **B.** Scatter plot of orientation and direction tuning strengths for orientation selective cells. **C.** Same as B but for direction selective cells. **D-F.** Same as A-C but using orientation selectivity index (OSI) and direction selectivity index (DSI) calculations. **G.** histograms depicting distribution of orientation selective cells (top row), direction selective cells (middle row), or all other cells (bottom row) based on different orientation or direction tuning calculations. N = 3259 DS cells and 7789 OS cells.

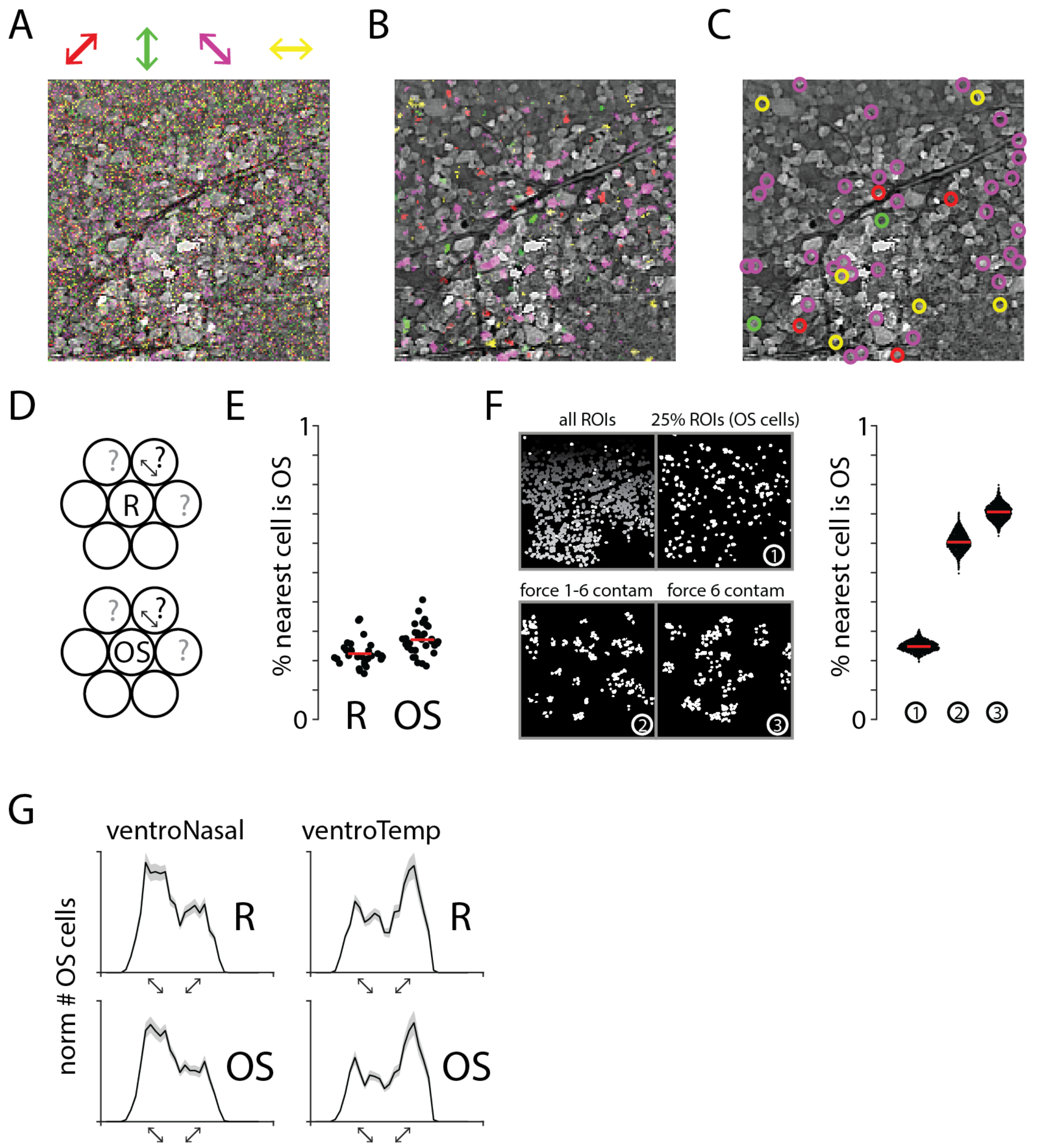

Supplemental Figure 2 – Overrepresentation of OS responses is not due to cross contamination of signal from nearby cells.

A. Pixel-based analysis using methods from X and Y. Briefly, the color represents the preferred orientation calculated for each pixel, and the hue represents the OS tuning (L_{ori}). **B.** To account for individual pixel noise in A, we applied a 2D median filter (2x2) to remove solitary pixels and

retain clusters of similar-orientation-preference pixels that are putative OS cell. **C.** Statistically-significant OS cells from our analysis map well on this pixel-based map though some discrepancies remain, likely due to the fact that our analysis uses both a quality index threshold (cells must have consistent responses to repeated trials) and a permutation test to determine statistically-significant OS cells. **D.** Analysis to test if cross-contamination of ROIs exists in our analysis. We identified all the OS cells in the normal-reared dataset and examined the 6 nearest neighbors of each OS cell (we chose 6 because a hexagonal matrix provides the best packing factor and is similar to the packing of cells in the ganglion cell layer). We performed the same analysis for a samples-matched number of randomly selected cells (R). **E.** There are more OS cells neighboring OS cells in our dataset (mean±stdev = 30.62±3.0%) than random cells (mean±stdev = 25.44±2.7%), but this difference is small and far from the numbers expected from modeled data of cross contamination (see panel F). **F.** Modeling to test how cross-contamination would affect analysis in D. We used a sample mask of ROIs from our dataset ("All ROIs") and simulated three distributions of cells with varying degrees of cross-contamination. First (1), we randomly sampled 25% of ROIs to be OS, resulting in a distribution that does not force cross-contamination. Second (2), we forced varying levels of cross-contamination (1-7 neighbors per cluster of ROIs) while still making sure that OS cells accounted for 25% of ROIs. Third (3), we repeated step two but now forced every cluster to include 7 ROIs. We repeated the calculations in D-E for 1000 simulations to obtain the distributions found at right in F (mean±stdev: 1: 24.87±1.8%; 2: 60.40±3.1%; 3: 70.60±2.7%). The random sampling of OS cells (1) resulted in values similar to our dataset whereas forcing cross-contamination greatly increased the likelihood that neighboring ROIs are OS. **G.** Of the neighboring OS cells found in either the OS or R dataset, we observe the same distributions of preferred orientations in both ventronasal and ventrotemporal retina.

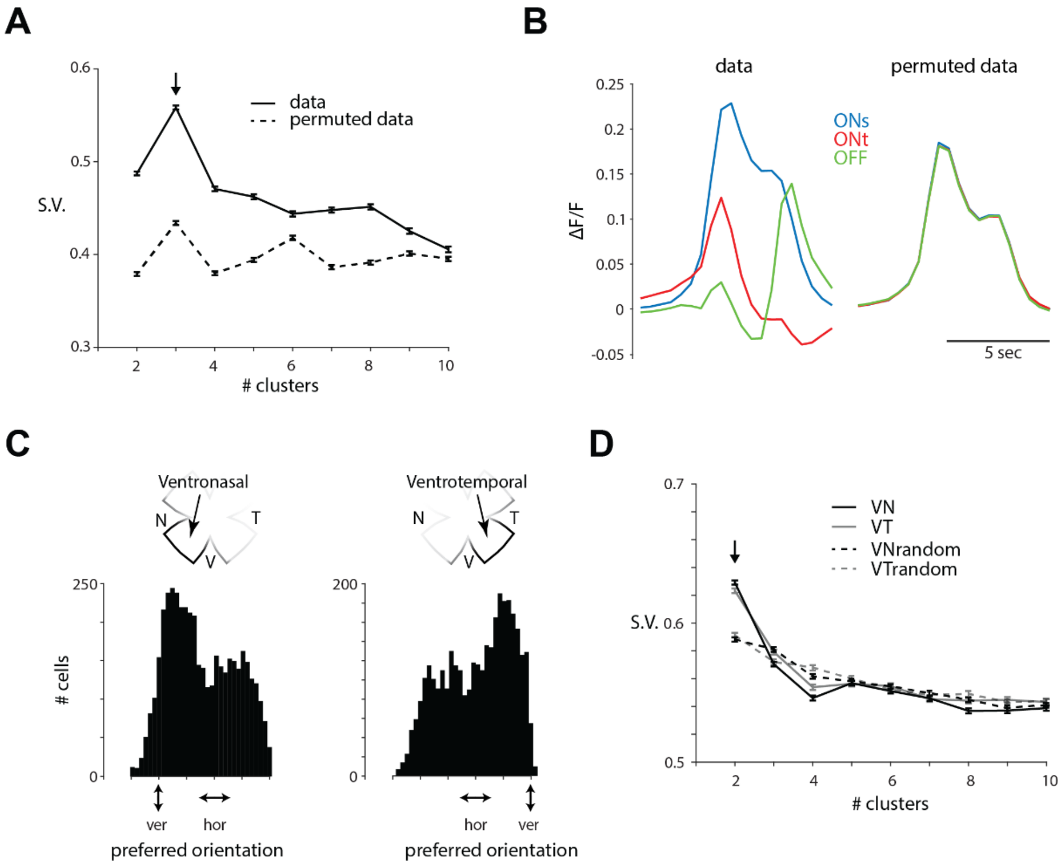

Supplemental Figure 3 – Functional clustering of OS cells based on direction and ON/OFF response.

A. Silhouette analysis of OS cells' responses to onset and offset of light reveals that the distribution of OS cells most segregates into three clusters (arrow). The dotted line represents a silhouette analysis where the responses of OS cells to light onset and offset were randomly permuted among each other. **B.** Average traces of the 3 clustered functional groups that were segregated using the silhouette analysis on the actual data (left) or the permuted data (right). **C.** Histograms of preferred orientations of statistically significant OS cells in ventronasal (left) and ventrotemporal (right) retina. **D.** Silhouette analysis of preferred orientations reveals that the distribution most segregates into two clusters (arrow). VNrandom and VTrandom represent silhouette analysis where the preferred orientations were shuffled 90° around the temporal direction (which is the center of both distributions).

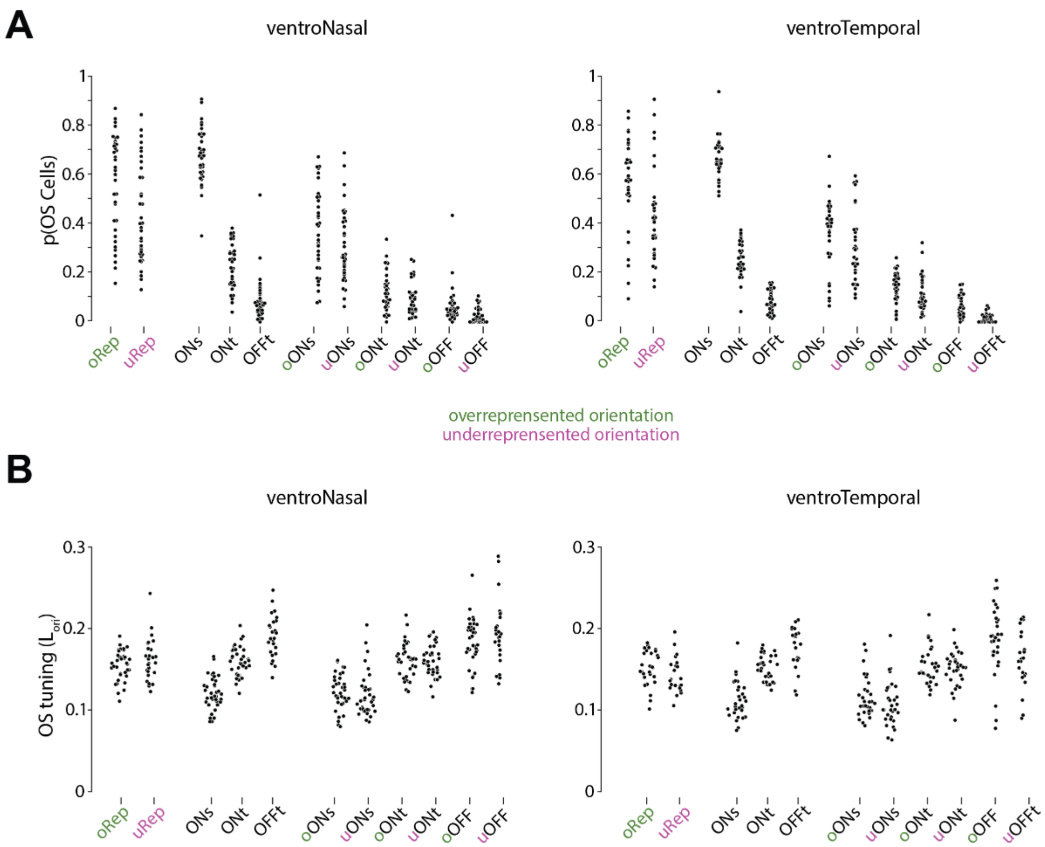

Supplemental Figure 4 – Proportion and tuning properties of orientation selective cells is constant throughout retinal space.

A. Proportion of OS subtypes among all OS cells only in ventroNasal (left) and ventroTemporal (right) retina. “oRep” direction refers to the overrepresented preferred orientation, whereas “uRep” refers the underrepresented preferred orientation. Each data point represents the proportion of a cell’s subtype within one FOV. **B.** Same as A but comparing tuning strength (L_{ori}) of all OS subtypes in ventroNasal (left) and ventroTemporal (right) retina. Each data point represents the average of a subtype’s tuning strength within one FOV. N = 67 FOVs

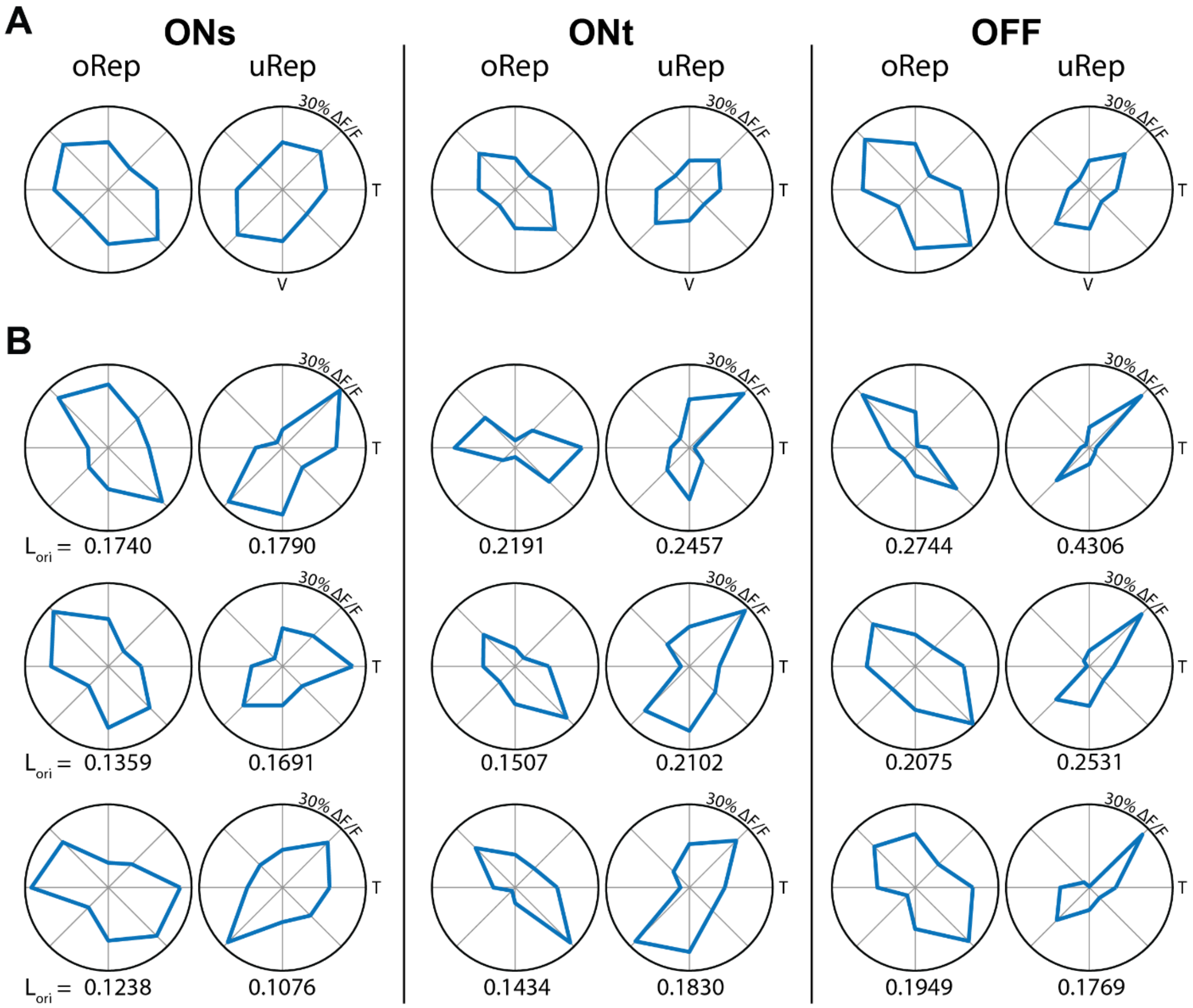

Supplemental Figure 5 – Average and example tuning curves of OS cell subtypes in ventronasal retina.

A. Average tuning curve of each of the 6 OS functional subtypes identified in our dataset. oRep: overrepresented preferred orientations, uRep: underrepresented preferred orientations. **B.** Tuning curves of 3 example cells for each of the 6 OS functional subtypes. The tuning strength, computed as L_{ori} is displayed at the bottom of each tuning curve.

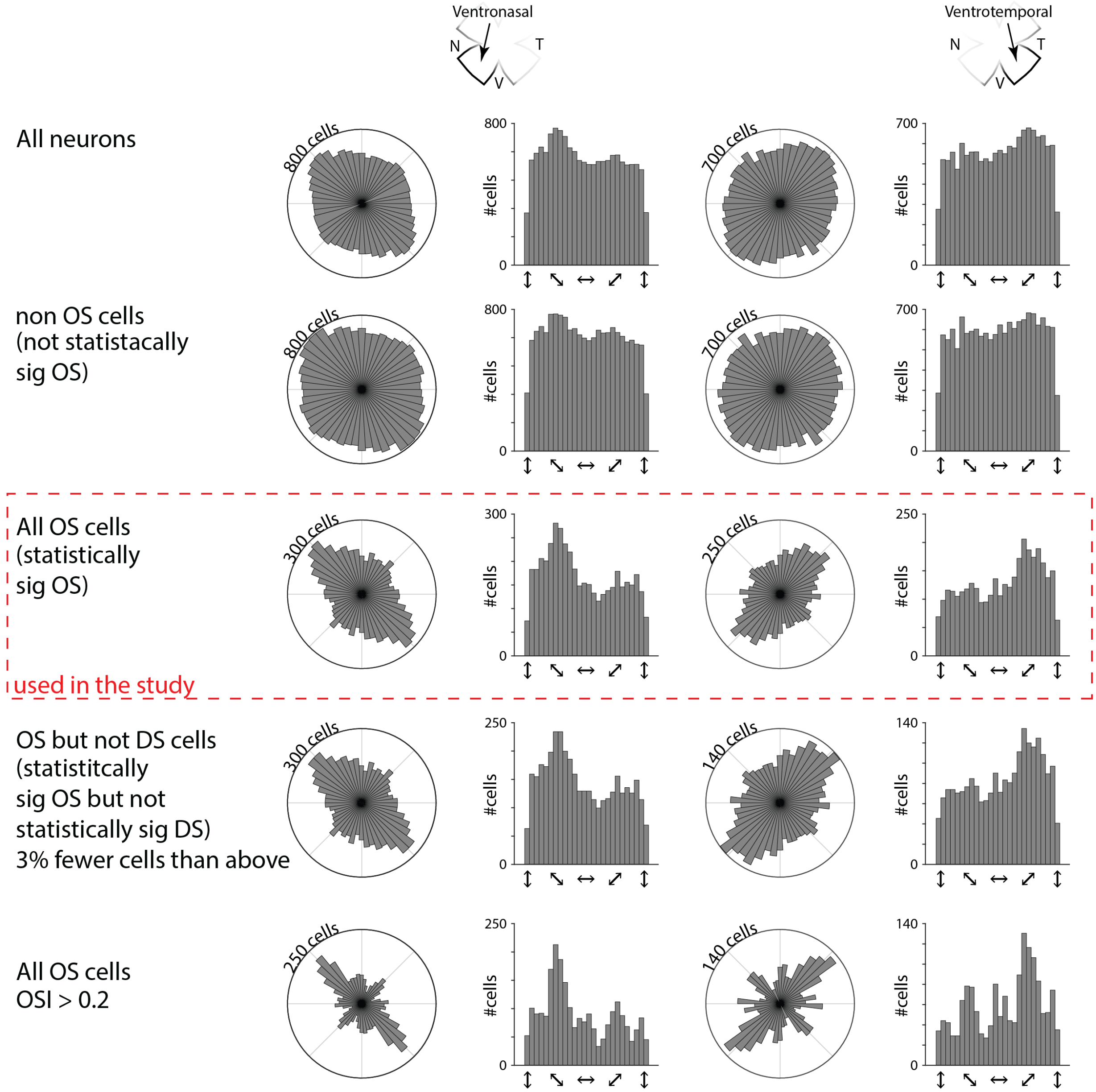

Supplemental Figure 6 – Mapping of OS cells does not dramatically change depending on criteria for identifying OS cells.

Mapping of the preferred orientation of cells in ventronasal (left columns) and ventrotemporal (right columns) retina. Each row represents different inclusion criteria. From top to bottom: 1st row: All neurons included. 2nd row: All neurons without the statistically significantly orientation selective neurons. 3rd row: All neurons that are statistically significantly orientation selective

(used in our study, see methods section for detailed analysis on how cells are determined to be statistically significantly orientation selective). 4th row: Same as 3rd row but removing 3% of orientation selective cells that are also statistically significantly direction selective cells. 5th row: Only cells that exhibit a greater orientation selectivity index (OSI) than 0.2 are included.

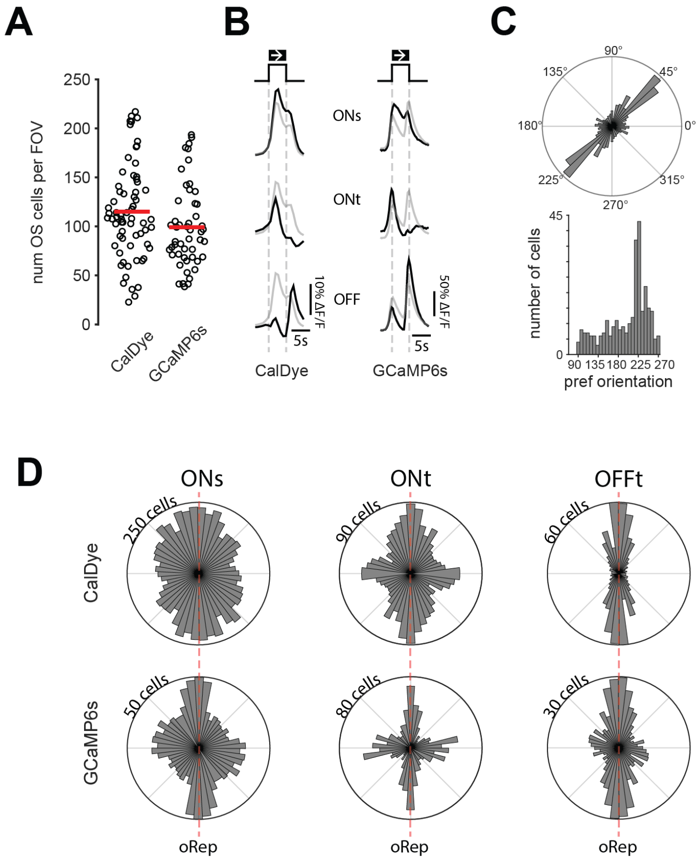

Supplemental Figure 7 – A dataset collected exclusively from retinal ganglion cells exhibits similar features as the calcium dye dataset.

A. Number of orientation selective cells per field of view (FOV) in the calcium dye and vglut2cre::GCaMP6s dataset (GCaMP6s only in retinal ganglion cells in the ganglion cell layer). Red bars represent the means. N = 50 FOVs across 4 mice. **B.** Average response of each of the three subtypes (ONs, ONt, and OFFt) to bars of moving light. **C.** Organization of preferred orientation in a subset of data collected in ventrotemporal retina in the vglut2cre::GCaMP6s mouse. N = 293 OS neurons across 4 fields of view in 1 mouse. **D.** Organization of preferred orientation anchored on the overrepresented orientation (red line) in the calcium dye (top row) and GCaMP6s dataset (bottom row).

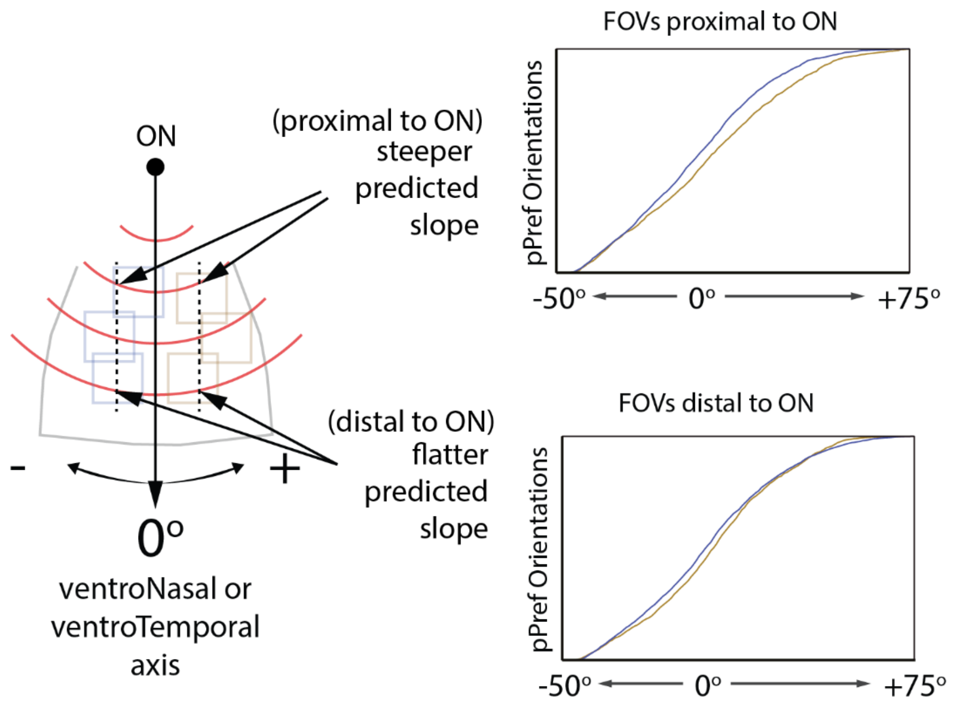

Supplemental Figure 8 – Greater deviations of preferred orientations from the nasotemporal axis in areas more proximal to the optic nerve.

Left: Schematic representation showing that predicted concentric circles have steeper slopes a set distance from the ventroNasal/ventroTemporal axis in FOVs proximal to the optic nerve (ON) than in FOVs distal to the ON. **Right:** Cumulative distributions showing greater deviations in the preferred orientations of OS cells in FOVs proximal to the ON (Top) than distal to the ON (Bottom).
