# Supplementary material for: Development and organization of the retinal orientation selectivity map
Source: Nat Commun. 2024 Jun 6;15:4829. doi: 10.1038/s41467-024-49206-z (PMC11156980; doi:10.1038/s41467-024-49206-z)
Supplement: Supplementary file 3 — Reporting Summary [file 41467_2024_49206_MOESM3_ESM.pdf]

nature portfolio

Corresponding author(s): Alexandre Tiriac
Last updated by author(s): May 20, 2024

Reporting Summary

Nature Portfolio wishes to improve the reproducibility of the work that we publish. This form provides structure for consistency and transparency in reporting. For further information on Nature Portfolio policies, see our Editorial Policies and the Editorial Policy Checklist.

Statistics

For all statistical analyses, confirm that the following items are present in the figure legend, table legend, main text, or Methods section.

n/a	Confirmed	
☐	☒	The exact sample size (*n*) for each experimental group/condition, given as a discrete number and unit of measurement
☐	☒	A statement on whether measurements were taken from distinct samples or whether the same sample was measured repeatedly
☐	☒	The statistical test(s) used AND whether they are one- or two-sided<br>*Only common tests should be described solely by name; describe more complex techniques in the Methods section.*
☐	☒	A description of all covariates tested
☐	☒	A description of any assumptions or corrections, such as tests of normality and adjustment for multiple comparisons
☐	☒	A full description of the statistical parameters including central tendency (e.g. means) or other basic estimates (e.g. regression coefficient) AND variation (e.g. standard deviation) or associated estimates of uncertainty (e.g. confidence intervals)
☐	☒	For null hypothesis testing, the test statistic (e.g. *F*, *t*, *r*) with confidence intervals, effect sizes, degrees of freedom and *P* value noted<br>*Give P values as exact values whenever suitable.*
☒	☐	For Bayesian analysis, information on the choice of priors and Markov chain Monte Carlo settings
☒	☐	For hierarchical and complex designs, identification of the appropriate level for tests and full reporting of outcomes
☒	☐	Estimates of effect sizes (e.g. Cohen's *d*, Pearson's *r*), indicating how they were calculated

Our web collection on statistics for biologists contains articles on many of the points above.

Software and code

Policy information about availability of computer code

Data collection	We use the data that was collected for Tiriac et al., 2022, doi: 10.1016/j.celrep.2021.110225<br>MATLAB R2022a<br>ScanImage<br>FIJI (ImageJ 1.54F)<br>Simple Neurite Trace FIJI plugin<br>G*Power
Data analysis	MATLAB R2022a.<br>Cellpose 2.0<br>FIJI (Fiji is just imageJ)<br>G*Power<br>Custom written MATLAB code available on the project GitHub page: https://github.com/TiriacLabCodeShare/OSproject

For manuscripts utilizing custom algorithms or software that are central to the research but not yet described in published literature, software must be made available to editors and reviewers. We strongly encourage code deposition in a community repository (e.g. GitHub). See the Nature Portfolio guidelines for submitting code & software for further information.

Data

Policy information about availability of data

All manuscripts must include a data availability statement. This statement should provide the following information, where applicable:

- Accession codes, unique identifiers, or web links for publicly available datasets
- A description of any restrictions on data availability
- For clinical datasets or third party data, please ensure that the statement adheres to our policy

Processed data is available on our lab's Github page: https://github.com/TiriacLabCodeShare/OSproject. The non-processed dataset is available upon request.

Research involving human participants, their data, or biological material

Policy information about studies with human participants or human data. See also policy information about sex, gender (identity/presentation), and sexual orientation and race, ethnicity and racism.

Reporting on sex and gender	*Use the terms sex (biological attribute) and gender (shaped by social and cultural circumstances) carefully in order to avoid confusing both terms. Indicate if findings apply to only one sex or gender; describe whether sex and gender were considered in study design; whether sex and/or gender was determined based on self-reporting or assigned and methods used. Provide in the source data disaggregated sex and gender data, where this information has been collected, and if consent has been obtained for sharing of individual-level data; provide overall numbers in this Reporting Summary. Please state if this information has not been collected. Report sex- and gender-based analyses where performed, justify reasons for lack of sex- and gender-based analysis.*
Reporting on race, ethnicity, or other socially relevant groupings	*Please specify the socially constructed or socially relevant categorization variable(s) used in your manuscript and explain why they were used. Please note that such variables should not be used as proxies for other socially constructed/relevant variables (for example, race or ethnicity should not be used as a proxy for socioeconomic status). Provide clear definitions of the relevant terms used, how they were provided (by the participants/respondents, the researchers, or third parties), and the method(s) used to classify people into the different categories (e.g. self-report, census or administrative data, social media data, etc.) Please provide details about how you controlled for confounding variables in your analyses.*
Population characteristics	*Describe the covariate-relevant population characteristics of the human research participants (e.g. age, genotypic information, past and current diagnosis and treatment categories). If you filled out the behavioural & social sciences study design questions and have nothing to add here, write "See above."*
Recruitment	*Describe how participants were recruited. Outline any potential self-selection bias or other biases that may be present and how these are likely to impact results.*
Ethics oversight	*Identify the organization(s) that approved the study protocol.*

Note that full information on the approval of the study protocol must also be provided in the manuscript.

Field-specific reporting

Please select the one below that is the best fit for your research. If you are not sure, read the appropriate sections before making your selection.

☒ Life sciences ☐ Behavioural & social sciences ☐ Ecological, evolutionary & environmental sciences

For a reference copy of the document with all sections, see nature.com/documents/nr-reporting-summary-flat.pdf

Life sciences study design

All studies must disclose on these points even when the disclosure is negative.

Sample size	We used the data from Tiriac et al., 2022, doi: 10.1016/j.celrep.2021.110225. The sample sizes for that study were initially planned out using power analyses in G*power with alpha set at 0.05 and power at 0.8 and ultimately resulted in Ns that were much larger than previously published results (Bos et al., 2016).<br><br>For present study, sample sizes are reported in the figures and figure captions. Depending on the analysis, samples are either cells or fields of views (when comparing proportion of cells for example).<br><br>Sample sizes:<br>NR: 7760 orientation selective cells across 67 fields of view across 7 mice; DR: 10908 orientation selective cells across 81 fields of views across 7 mice; B2KO: 5452 orientation selective cells across 55 fields of view across 8 mice.
Data exclusions	Fields of views with unequal average responses to all light stimuli trials (3x8 direction, so 24) were excluded from the dataset. This was pre-established before we began to segregate the cells from the fields of views. Uneven responses to the 24 light presentations sometimes occured dif the z focus changed during acquisition or if a stray air bubble entered the FOV during acquisition.

Replication	We used large N (number of cells) to ensure replication. Though not intended as replication, our results from the DR and B2KO replicate the findings we see in the NR dataset.
Randomization	In the initial data acquisition study, males and females were randomly allocated to different experimental group and ultimately balanced. During segmentation of cells within each field of view, we randomly processed images from the different conditions to ensure that the segmentation was similar across groups.
Blinding	Experimenters were not blinded during data acquisition. Experimenters were blinded during the segmentation of cells (One of the first analysis steps when we identify all neurons within a field of view). All other analyses after the segmentation of neurons are performed on the entire dataset using the same algorithm and therefore does not require blinding.

Reporting for specific materials, systems and methods

We require information from authors about some types of materials, experimental systems and methods used in many studies. Here, indicate whether each material, system or method listed is relevant to your study. If you are not sure if a list item applies to your research, read the appropriate section before selecting a response.

Materials & experimental systems

n/a	Involved in the study
☒	☐ Antibodies
☒	☐ Eukaryotic cell lines
☒	☐ Palaeontology and archaeology
☐	☒ Animals and other organisms
☒	☐ Clinical data
☒	☐ Dual use research of concern
☒	☐ Plants

Methods

n/a	Involved in the study
☒	☐ ChIP-seq
☒	☐ Flow cytometry
☒	☐ MRI-based neuroimaging

Animals and other research organisms

Policy information about studies involving animals; ARRIVE guidelines recommended for reporting animal research, and Sex and Gender in Research

Laboratory animals	NR and DR dataset come from C57B6 mice, aged between 30-60 days old (Stock No: 000664). The b2-nAChR-KO mice were aged 30-60 days old and were bred in lab (Bansal et al., 2000). Vglut2-Cre::GCaMP6s mice were originally obtained from Jackson laboratories and bred in lab (vglut2cre Strain #:028863; gcamp6s Strain #:028866)
Wild animals	*Provide details on animals observed in or captured in the field; report species and age where possible. Describe how animals were caught and transported and what happened to captive animals after the study (if killed, explain why and describe method; if released, say where and when) OR state that the study did not involve wild animals.*
Reporting on sex	We ran analyses to check for sex-related differences but found none. For the paper, sexes were combined. Previous papers studying similar topics found no sex-related differences.
Field-collected samples	*For laboratory work with field-collected samples, describe all relevant parameters such as housing, maintenance, temperature, photoperiod and end-of-experiment protocol OR state that the study did not involve samples collected from the field.*
Ethics oversight	No ethical guidance required since we used data acquired from Tiriac et al, 2022. From original study: "All animal procedures were approved by the UC Berkeley Institutional Animal Care and Use Committee and conformed to the NIH Guide for the Care and Use of Laboratory Animals, the Public Health Service Policy, and the SFN Policy on the Use of Animals in Neuroscience Research"

Note that full information on the approval of the study protocol must also be provided in the manuscript.
